# Tricuspid Regurgitation and Impact of Surgical Valve Intervention in the Single Ventricle Reconstruction Trial

**DOI:** 10.1007/s00246-025-04122-x

**Published:** 2026-02-18

**Authors:** J. F. Cnota, S. M. Chowdhury, A. Floh, R. Gongwer, B. H. Goot, J. P. Jacobs, M. A. Jolley, S. Kirmani, D. J. LaPar, J. C. Levine, A. B. Lewis, R. H. Pignatelli, C. Pizarro, T. C. Slesnick, T. Thorsson, F. Trachtenberg, D. T. Truong, J. W. Newburger, P. C. Frommelt

**Affiliations:** 1 Department of Pediatrics, Cincinnati Children’s Hospital Medical Center, Heart Institute, 3333 Burnet Avenue, Cincinnati, OH 45244 USA; 2 Department of Pediatrics, Medical University of South Carolina, Charleston, SC USA; 3 Department of Pediatrics and Labatt Family Heart Centre, Hospital for Sick Children, University of Toronto, Toronto, Canada; 4 New England Research Institutes, Watertown, MA USA; 5 Department of Pediatrics, Herma Heart Institute, Children’s Wisconsin and Medical College of Wisconsin, Milwaukee, WI USA; 6 Division of Thoracic and Cardiovascular Surgery, Department of Surgery, University of Florida, Gainesville, FL USA; 7 Division of Pediatric Cardiology, Department of Anesthesia and Critical Care, Children’s Hospital of Philadelphia, Philadelphia, PA USA; 8 Department of Pediatrics, Duke University Medical Center, Durham, NC USA; 9 Morgan Stanley Children’s Hospital of New York Presbyterian Columbia University Medical Center and Department of Pediatrics, Division of Cardiology Columbia University, New York, NY USA; 10 Department of Cardiology, Boston Children’s Hospital and Department of Pediatrics, Harvard Medical School, Harvard University, Boston, MA USA; 11 Department of Pediatrics, Children’s Hospital Los Angeles, Los Angeles, CA USA; 12 Pediatric Cardiology, Baylor College of Medicine/Texas Children’s Hospital, Houston, TX USA; 13 Alfred I. DuPont Hospital for Children and Department of Pediatrics, Division of Cardiology Thomas Jefferson University, Wilmington, DE USA; 14 Department of Pediatrics, Emory University and Children’s Healthcare of Atlanta, Atlanta, GA USA; 15 Department of Pediatrics, Medical School, University of Michigan and Michigan Medicine, Ann Arbor, MI USA; 16 Department of Pediatrics, University of Utah and Primary Children’s Hospital, Salt Lake City, UT USA

**Keywords:** Tricuspid regurgitation, Hypoplastic left heart syndrome, Survival, Norwood procedure

## Abstract

Tricuspid regurgitation (TR) is common in single right ventricle (RV) anomalies and affects outcomes during staged surgical palliations. The Pediatric Heart Network’s (PHN) Single Ventricle Reconstruction trial followed children with single RV to age 6 years. This study compared serial echocardiographic measures of TR during staged palliations and determine the impact of TR and tricuspid valve (TV) intervention on transplant-free survival in the PHN cohort. Study echocardiograms were performed at 6 pre-determined times: baseline, post-Norwood, pre-stage II, age 14 months, pre-Fontan, and age 6 years. TR was categorized as *≤* mild or > mild. McNemar’s test compared TR across time; t-tests compared RV size/function by TR category; and survival analysis assessed the effect of > mild TR on transplant-free survival. TR was assessed in 543 participants prior to Norwood. From baseline to post-Norwood, those with > mild TR increased from 12 to 24% (*p* < 0.001). Thereafter, the proportion of surviving participants with > mild TR was stable. Participants with > mild TR had increased baseline TV annular area, and increased RV volume but not decreased function. Transplant-free survival at 6 years was lower with > mild TR at any time except pre-Fontan. 78 TV interventions occurred in 66 participants. Those with TV intervention at the Norwood and/or Stage II had a higher rate of death/transplant than those without or those who had a later intervention. At baseline, post Norwood and pre-stage II, > mild TR is associated with decreased transplant-free survival. TV intervention at the Norwood or Stage II operations was not associated with improved survival.

Clinical Trial Registration: NCT00115934

**Supplementary Information:**

The online version contains supplementary material available at https://doi.org/10.1007/s00246-025-04122-x.

## Introduction

Tricuspid valve (TV) regurgitation is relatively common in patients with single right ventricle (RV) anomalies and affects outcome during staged surgical palliations. A greater amount of tricuspid regurgitation (TR) is associated with decreased survival and higher rates of cardiac transplantation [1, 2]. Anatomic and functional mechanisms contributing to the development of TR include tethering of the septal leaflet, cleft leaflets, annular dilation, and leaflet prolapse [3–6]. Despite the clinical significance of the problem and advances in understanding the pathophysiology, effective surgical management strategies remain elusive and long-term outcomes suboptimal [7, 8]. 

The Single Ventricle Reconstruction (SVR) trial compared outcomes of infants with single RV anomalies who underwent Norwood procedure and were randomized to either a modified Blalock-Taussig shunt or right ventricle-to-pulmonary artery shunt as the source of pulmonary blood flow [9]. The cohort has been followed longitudinally with overall transplant-free survival of 61% at 6 years-of age and no significant difference by shunt type [10]. Moderate or greater TR prior to Norwood was associated with worse transplant-free survival at 6 years-of-age (HR 1.77; 95% CI 1.23–2.56). Echocardiographic assessment in survivors at 6 years of age suggested favorable RV remodeling with decreased ventricular and TV size and improved systolic and diastolic function [11]. However, this analysis did not include detailed assessment of TR severity over time or the effect of TR on outcomes after each staged palliation.

In this analysis of the SVR study cohort, we sought to address the following aims: (1) determine if the degree of neonatal TR prior to Norwood is associated with the degree of TR at later time points, (2) assess for change in the degree of TR following each surgical palliative stage to age 6 years, (3) determine the association between surgical tricuspid intervention on TV function and survival to age 6 years, (4) serially compare associations between RV size and function and tricuspid annular size with the prevalence of > mild TR, and (5) determine the association between the degree of TR and death/transplant at each time point.

## Materials and Methods

### Participants

The enrollment criteria for the SVR trial have been previously published [12]. This analysis included data from SVR participants who underwent the Norwood procedure and had at least one echocardiogram deemed acceptable for analysis up to age 6 years. Participants who underwent cardiac transplantation were censored and those who had conversion to biventricular physiology were excluded.

### Echocardiographic Analysis

Protocol study echocardiograms were performed at 6 pre-determined times: baseline prior to any interventions, early post-Norwood at a mean age 22.5 ± 13.4 days, pre-stage II at a mean age 4.8 ± 1.8 months, age 14 months, pre-Fontan at mean age of 34.3 ± 10.4 months, and post-Fontan at age 6 years. The 2D/Doppler echocardiograms were analyzed by a core laboratory at the Medical College of Wisconsin as previously described [11, 13]. TR was categorized as none, mild, moderate, and severe based on qualitative assessment of the regurgitant jet vena contracta size using color Doppler, then dichotomized as *≤* mild or > mild for analysis. The degree of TR was assessed at each time point, independent of prior severity or intervention. Calculation of tricuspid annular area was performed when measures were available, but TV anatomic variables were not assessed.

### Statistical Analysis

Summary descriptive statistics are presented for demographics and echocardiographic indices. Categorical variables were summarized by frequency and percentage. Continuous variables were summarized as mean ± standard deviation, or median and range as appropriate. McNemar’s Exact Test was used to compare TR category between one echocardiogram and the next echocardiogram, as well as between baseline and other time points. T-tests were used to compare RV size and systolic function by TR category. Fisher’s exact tests were used to compare rates of death/transplant through 6 years by TR category. Survival analysis was used to assess the effect of > mild TR on transplant-free survival. These analyses were stratified by the degree of TR at the different time points: baseline, post-Norwood, pre-Stage II, 14-month, and pre-Fontan. Survival analysis was similarly used to model the association between TV intervention and transplant-free survival. The proportional hazards assumption was tested for each model with the Supremum Test for proportional hazards. When the proportional hazard assumption was violated, cut-points for pre/post Stage II and pre/post Fontan were used as previously applied for the cohort’s 6-year main results paper [10]. Because both TR and AV valve intervention are time-dependent and the risk of temporal bias is strong, a Cox model with time-varying covariates (TR severity and AV valve intervention) was also applied to the outcome of death/transplant.

## Results

Of the 920 patients at eligibility screening, a baseline echocardiogram was performed in 555 participants. Of these, 549 had the Norwood procedure, and 3 were excluded for subsequent conversion to biventricular repair. Most participants had echocardiograms that were acceptable for analysis, therefore the baseline cohort for this analysis consisted of 543 participants. Table 1 describes the frequency of echocardiograms and TR determinations for each of the prespecified time points.

**Table 1:** Frequency of echocardiograms and TR by time point

Echocardiogram	*N*	*n* (%) with TR determination	*n* (%) with > mild TR	*n* (%) with > mild TR, among those with a 6-year echo
Baseline	532	521 (97.9)	63 (12.1)	20 (8.7)
Study Visit 1 (post Norwood)	471	466 (98.9)	113 (24.3)	54 (23.1)
Study Visit 2 (pre-Stage II)	388	386 (99.5)	102 (26.4)	54 (23.1)
Study Visit 4 (14 months)	325	321 (98.8)	71 (22.1)	44 (19.9)
Pre-Fontan Echo	256	255 (99.6)	46 (18.0)	39 (19.3)
6 Year Echo	235	235 (100)	41 (17.5)	41 (17.5)

### TR Severity in Transplant-Free Survivors to Age 6-Years

When compared to the baseline echocardiogram, there was a significant increase in the proportion of participants with > mild TR at the post-Norwood time point (12.1% vs. 24.3%, *p* < 0.001). The proportion with > mild TR on subsequent echocardiograms remained stable to 6 years of age (Table 1) and was unchanged when the analysis cohort was defined in the following three ways: (1) all available participants, (2) participants with adequate echocardiogram at the 6-year time point, or (3) participants with adequate echocardiograms at all 6 time points. Among all participants, 54% had *≤* mild TR at all available echocardiograms (50% among transplant-free survivors), and 4% had > mild TR at all available echocardiograms (1% among transplant-free survivors). Among the 186 participants with all 6 echocardiograms, 49% had *≤* mild TR across all 6 echocardiograms, and only 2 participants (1.1%) had consistently > mild TR.

### Association of Right Ventricular Size/Function with TR Severity

Echocardiographic measures of RV size and function were presented by TR category in Table 2. At the post-Norwood time point, participants with *≤* mild TR had slightly higher ejection fraction (47% vs. 45%, *p* = 0.005) and fractional area change (37% vs. 35%, *p* = 0.02) than those with > mild TR. However, this finding was modest and not seen at any other time point, as baseline TR severity was not consistently associated with later measures of RV size/function in survivors.

**Table 2:** Summary statistics of echo visits and parameters by TR status at echo (*N* = 532)

Echo Measure	TR group	Baseline		Study Visit 1 (post Norwood)		Study Visit 2 (pre-Stage II)		Study Visit 4 (14 Months)		Pre-Fontan Echo		6 Year Echo	
		*N*	Mean ± SD Median (Q1, Q3)	*N*	Mean ± SD Median (Q1, Q3)	*N*	Mean ± SD Median (Q1, Q3)	*N*	Mean ± SD Median (Q1, Q3)	*N*	Mean ± SD Median (Q1, Q3)	*N*	Mean ± SD Median (Q1, Q3)
RV ejection fraction	None/Mild TR	313	46.44 ± 8.65	279	47.20 ± 7.96	204	44.00 ± 8.06	179	43.26 ± 7.54	120	43.46 ± 6.95	92	45.83 ± 6.10
			46.44 (41.38, 51.88)		47.40 (42.24, 52.97)		43.68 (39.10, 49.98)		42.30 (38.91, 48.56)		43.53 (38.56, 48.22)		46.34 (41.68, 50.11)
	> Mild TR	31	45.06 ± 9.03	79	44.38 ± 7.45	69	43.67 ± 8.86	50	41.68 ± 8.01	30	43.38 ± 5.97	23	44.49 ± 6.90
			46.65 (41.93, 52.00)		45.11 (39.73, 49.08)		44.61 (36.35, 49.24)		43.18 (36.39, 46.26)		43.18 (39.77, 47.37)		43.35 (39.90, 50.00)
		*P* = 0.3968		**P = 0.0051**		*P* = 0.7727		*P* = 0.1972		*P* = 0.9565		*P* = 0.3575	
Indexed RV End-diastolic volume, mL/BSA^1.3^	None/Mild TR	314	86.01 ± 24.40	279	90.14 ± 25.89	203	110.36 ± 32.44	179	86.59 ± 24.16	120	89.03 ± 24.99	92	80.71 ± 19.05
			83.85 (68.74, 98.55)		87.18 (73.58, 102.21		106.65 (87.83, 125.7		84.97 (70.41, 99.65)		86.54 (70.93, 101.96		78.89 (65.51, 91.41)
	> Mild TR	31	94.84 ± 28.77	79	100.96 ± 27.80	69	122.95 ± 38.61	50	104.04 ± 41.07	30	106.14 ± 24.95	23	105.35 ± 33.43
			87.51 (76.54, 114.90		100.97 (82.45, 120.3		120.53 (93.21, 141.2		97.35 (77.40, 115.03		96.53 (88.32, 115.54		96.79 (78.84, 115.98
		*P* = 0.0597		**P = 0.0014**		**P = 0.0086**		**P = 0.0057**		**P = 0.0010**		**P = 0.0022**	
Indexed tricuspid valve annular area/BSA	None/Mild TR	426	6.06 ± 1.76	336	6.89 ± 1.88	274	6.68 ± 1.93	246	6.13 ± 1.87	193	5.43 ± 1.68	179	5.60 ± 1.42
			5.83 (4.74, 6.96)		6.92 (5.54, 8.09)		6.51 (5.43, 7.86)		5.99 (4.81, 7.24)		5.22 (4.31, 6.36)		5.59 (4.73, 6.45)
	> Mild TR	57	7.16 ± 2.04	108	7.55 ± 2.28	98	8.60 ± 3.16	69	7.46 ± 2.23	43	6.81 ± 1.68	41	6.73 ± 1.86
			7.00 (5.65, 8.58)		7.05 (5.93, 8.92)		8.08 (6.25, 10.30)		6.95 (5.92, 8.63)		6.70 (5.54, 8.01)		6.59 (5.37, 7.93)
		**P < 0.0001**		**P = 0.0072**		**P < 0.0001**		**P < 0.0001**		**P < 0.0001**		**P < 0.0006**	

All p-values are t-tests (Pooled or Satterthwaite as appropriate for equality of variance)

P-values < .05 are highlighted in bold

Indexed RV size, measured by indexed end-diastolic volume or area, and indexed tricuspid annular area were greater in participants with > mild TR, compared to those with *≤* mild TR, at most time points after the Norwood procedure. Baseline > mild TR was also associated with tricuspid annular dilatation present at birth, and this dilatation persisted until stage II palliation (for the entire cohort and for those who never had an AVV intervention). Similarly, > mild TR post Norwood was associated with tricuspid annular dilatation at birth that persisted to stage II palliation; those with > mild TR also had increased RV dilatation and poorer RV systolic function through the interstage period. RV size and TV size were dramatically decreased following the change in physiology after stage II palliation, regardless of the degree of TR.

### Association of TR Severity and Transplant-Free Survival

**Fig. 1:**
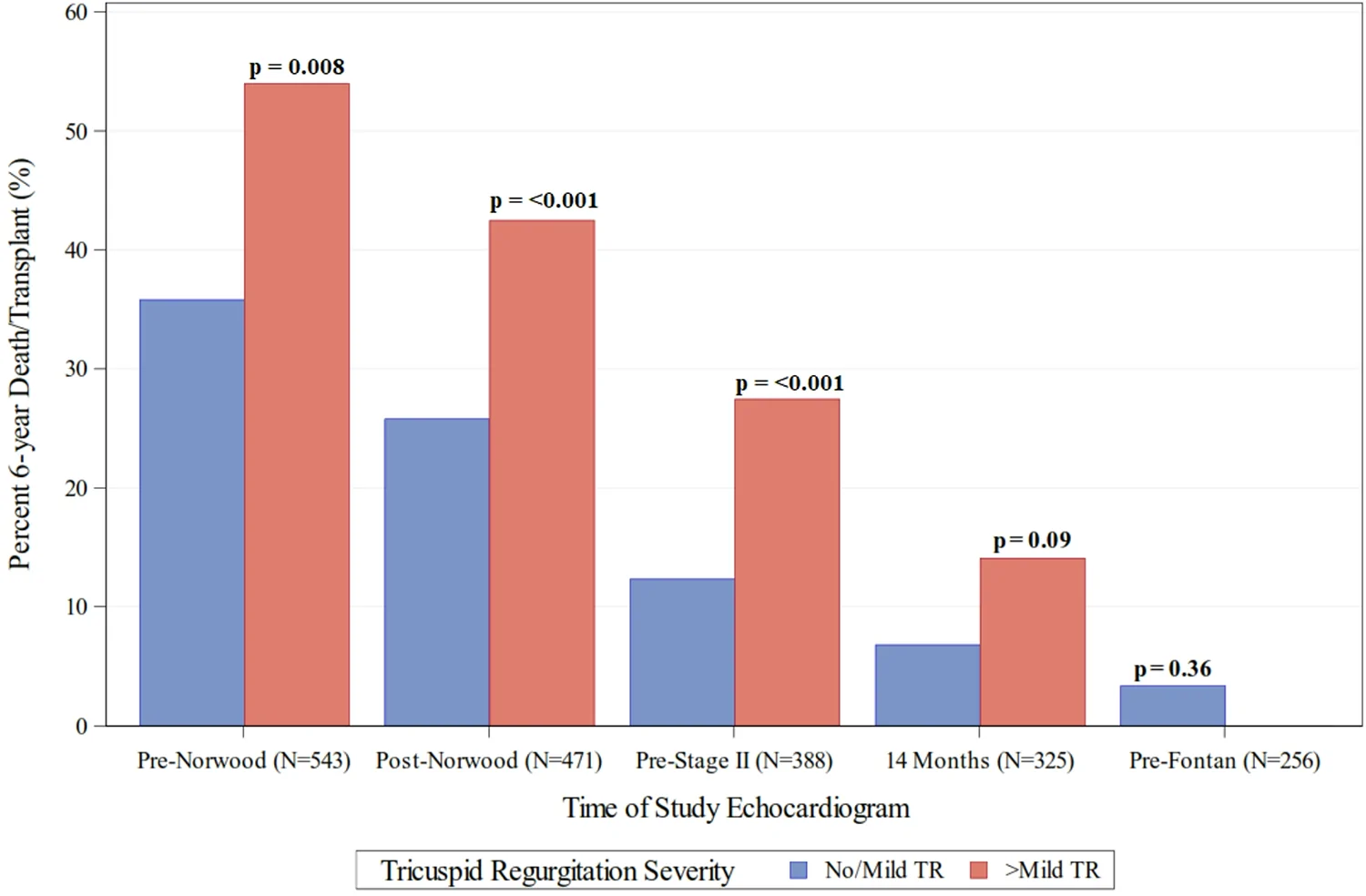
Death/Transplant through 6 years by TR severity at each study echocardiogram

**Fig. 2:**
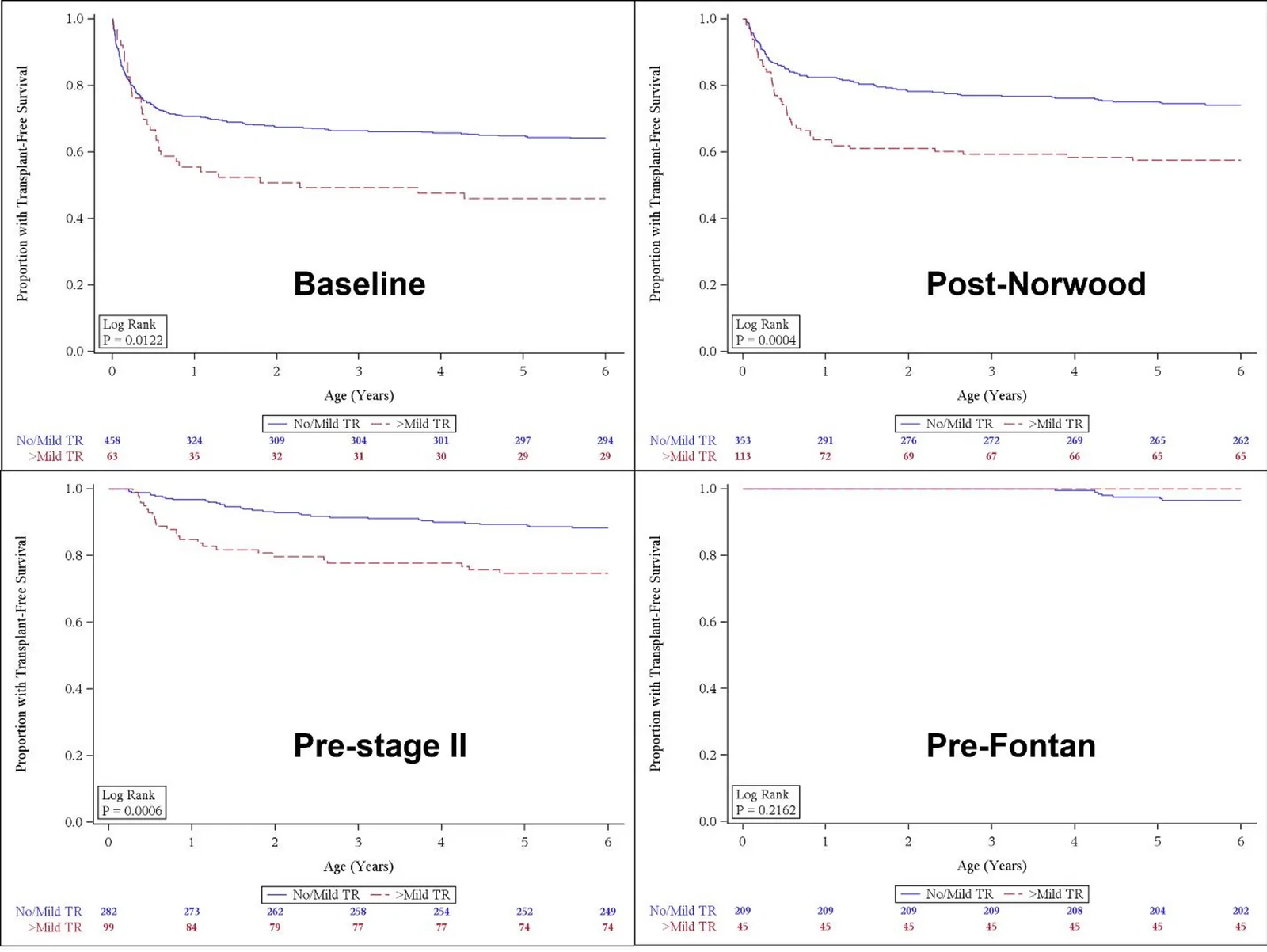
Probability of survival by TR severity on echocardiogram at 4 time points: baseline, post-Norwood, pre-Stage II, and pre-Fontan

Figure 1 compares the percentage of participants with death/transplant through 6 years by the TR severity at each study echocardiogram. In all intervals but the pre-Fontan echocardiogram, > mild TR was associated with death/transplant after that echocardiogram. Survival curves for transplant-free survival through 6 years were presented in Fig. 2. Each curve is stratified by TR severity at one of the prespecified echocardiogram time points.

### Characteristics of TV Interventions

A total of 78 TV interventions occurred in 66 unique participants. Most interventions were concurrent to staged palliation (9 at Norwood, 35 at Stage II, 27 at Fontan). However, a small proportion occurred at separate time points (1 during interstage, 6 following Fontan). Twelve participants had 2 interventions. Operative techniques used for tricuspid valvuloplasty were not available from the data collected, but three participants underwent AV valve replacement. Of the 9 who underwent AVV intervention at Norwood, none of the two with 6-year echos were *≤* mild. Of the 35 who underwent AVV intervention at Stage 2, 7/15 with 6-year echos were *≤*mild. Of the 21 who underwent AVV intervention at stage III, 4/18 with 6-year echo were *≤* mild.

### Association of AV Valve Intervention for TR and Transplant-Free Survival

**Fig. 3:**
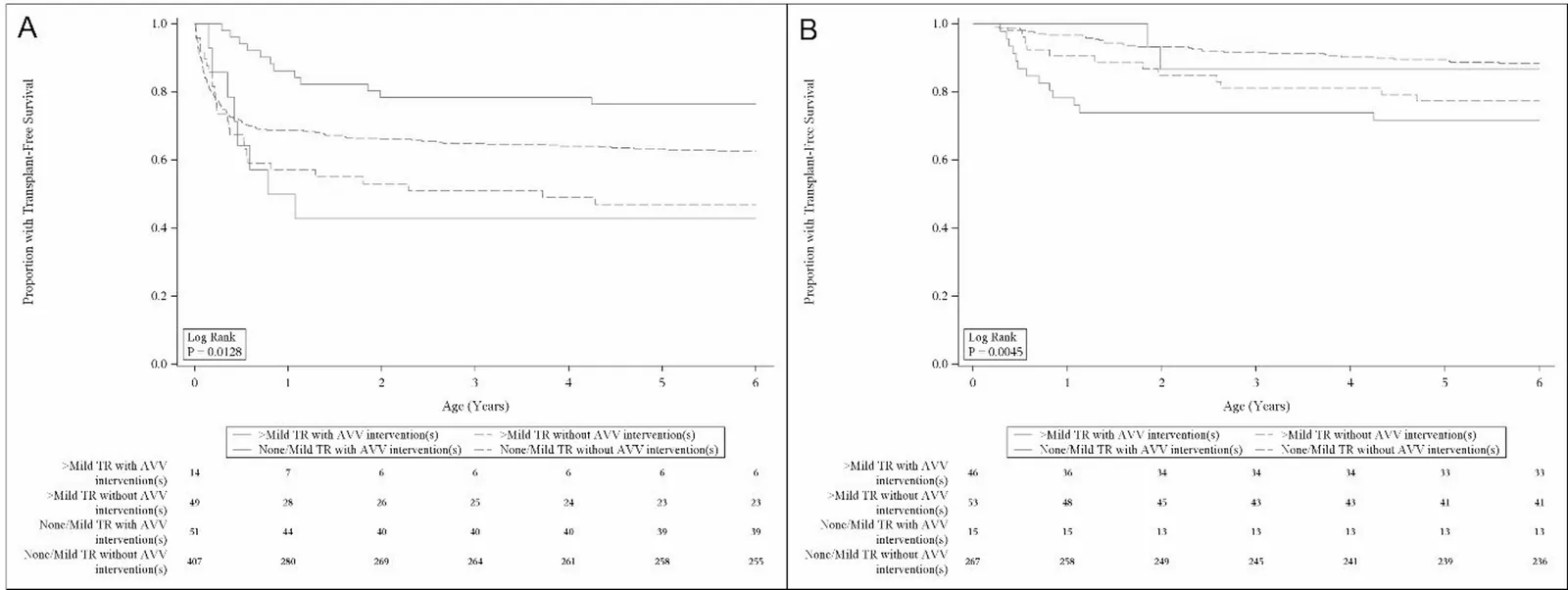
Probability of survival by TV intervention and stratified by TR severity at baseline echocardiogram (**A**) and pre-stage II echocardiogram (**B**)

Overall, transplant-free survival among those who did and did not undergo AVV intervention was not significantly different. However, transplant-free survival was worse if AVV intervention was performed at the Norwood or Stage II surgeries. The stratified survival curves in Fig. 3 do not demonstrate a survival advantage associated with AVV intervention. The Cox model with time-varying covariates (TR severity and AVV intervention) identifies increased death/transplant associated with > mild TR (HR 1.5, 95%CI, 1.1–2.1) and AVV intervention (HR 2.7, 95%CI, 1.6–4.6). AVV intervention at Norwood was associated with poorer transplant-free survival after the intervention, with heart transplant occurring in 33% of those following intervention and 3% of those without intervention, and persistence of > mild TR at subsequent echocardiograms. AVV intervention at Stage II was associated with progressive TR prior to the procedure from baseline to post-Norwood to pre-Stage II, and worse transplant-free survival after the intervention, with death/transplant in 30% compared to 10% with no intervention at that stage. TR was > mild in 90% of the 35 patients undergoing AVV intervention at stage II. Follow-up of transplant-free survivors at subsequent time points demonstrated: TR > mild in 12/21 (57%) at 14-months (*p* = 0.11 vs. stage II), 5/19 (26%) at pre-Fontan (*p* = 0.12 vs. 14-months), and 4/15 (27%) at 6-years (*p* = 0.5 vs. pre-Fontan). In contrast, AVV intervention at Fontan was associated with transplant-free survival of 95% and significantly decreased TR at 6-year follow-up compared to the pre-Fontan study.

## Discussion

This study demonstrated that > mild TR, when assessed at baseline and subsequent time points in early childhood, is consistently associated with worse transplant-free survival following the Norwood procedure and stage II palliation for single right ventricle anomalies. While the risk associated with TR has been associated with the baseline echocardiogram for the SVR cohort [10, 14], this study demonstrated that similar risk persists through the 14-month echocardiogram. While > mild TR at the pre-Fontan echocardiogram was not associated with subsequent transplant-free survival, this must be interpreted cautiously as the follow-up time was limited. In fact, recent long-term follow-up studies of the Fontan population suggest that TR remains a poor prognostic marker over time [15].

The proportion of children with > mild TR increased significantly from baseline to post-Norwood echocardiograms. There are a variety of possible explanations for this observation. The increased volume load with annular dilation [6] associated with postnatal physiology, myocardial injury associated with cardiopulmonary bypass, morphologically abnormal leaflets [5] or even direct injury to the TV apparatus are potential explanations. However, this increase in TR appears to stabilize after the post-Norwood echocardiogram. This may be, in part, attributable to survival bias, which reduces the number of individuals with > mild TR over time. Alternatively, the myocardium may recover from the early injury, and the survivors of TR repair have improved valve function. However, our results did not support a relationship between TR severity and RV systolic function. The decreased indexed RV and tricuspid annular size noted at the 14-month echocardiogram was subsequently maintained over time, reflecting the reduced volume load on the single RV following stage II palliation. Yet these changes in size were not associated with a change in TR severity, lending credence to the observation that TR is multifactorial, related to both physiologic and anatomic challenges that can be difficult to completely discern.

### Limitations

Our analysis was unable to demonstrate that TV intervention improves transplant-free survival in the setting of TR. In fact, the time-varying model suggests an increased hazard of death/transplant associated with TR intervention. The interpretation of the survival analysis for TV intervention in this cohort is particularly challenging with this data set and study design, as clinical and anatomic factors that drive the decision to intervene for TR [16–18] were not available for review. Confounding by indication may be present when the severity of TR is both an indication for TV intervention and a risk factor for death/transplant without intervention. With those considerations in mind, our findings are presented to highlight the need for ongoing efforts to improve valve management in patients with single RV anomalies [7, 18]. 

Further limitations include that the echocardiographic data are impacted by the standard geometric challenges in quantifying right ventricular size and function. Image and measurement quality were optimized by standard training of clinical sites and core laboratory protocols. TR was assessed qualitatively using color Doppler because this was the most robust tool available in the cohort, with more quantitative measures (such as vena contracta size/area) much less consistently available. Because of the association between TR severity and survival, the longitudinal assessment of TR could be subject to survivor bias. The small number of cardiac transplantations did not allow for detailed analysis of that subgroup.

## Conclusions

Greater than mild TR at baseline, post Norwood or prior to stage II is associated with decreased transplant-free survival in infants and children with single RV anomalies. While TV function worsens initially after the Norwood procedure, it then stabilizes in survivors, unrelated to changes in RV and TV size over time. TV intervention at the Norwood and stage II surgery is associated with worse transplant-free survival, but TV intervention at the Fontan procedure was associated with excellent transplant-free survival and a reduced TR. Since indications and anatomic features that led to the decision to intervene were unavailable, these findings may be a marker of disease severity rather than failed management and should be interpreted with caution. This study highlights the need for ongoing efforts to optimize valve function in children with single RV anomalies.

## Supplementary Information

Below is the link to the electronic supplementary material.

Supplementary Material 1

## Data Availability

No datasets were generated or analysed during the current study.

## Abbreviations

TR: Tricuspid regurgitation
PHN: Pediatric Heart Network
TV: Tricuspid valve
RV: Right ventricle
AVV: Atrioventricular valve
SVR: Single Ventricle Reconstruction
