# Supplementary material for: Tricuspid Regurgitation and Impact of Surgical Valve Intervention in the Single Ventricle Reconstruction Trial
Source: Pediatr Cardiol. 2026 Feb 18;47(8):3354–61. doi: 10.1007/s00246-025-04122-x (PMC13687309; doi:10.1007/s00246-025-04122-x)
Supplement: Supplementary file 1 [file 246_2025_4122_MOESM1_ESM.docx]

| Supplementary Table 1: Summary statistics of echo visits and parameters by TR status at echo (N = 532) | | | | | | | | | | | | | |
| --- | --- | --- | --- | --- | --- | --- | --- | --- | --- | --- | --- | --- | --- |
| **Echo Measure** | **TR group** | **Baseline** | | **Study Visit 1**  **(post Norwood)** | | **Study Visit 2 (pre-Stage II)** | | **Study Visit 4**  **(14 Months)** | | **Pre-Fontan Echo** | | **6 Year Echo** | |
|  |  | **N** | **Mean ± SD Median (Q1, Q3)** | **N** | **Mean ± SD Median (Q1, Q3)** | **N** | **Mean ± SD Median (Q1, Q3)** | **N** | **Mean ± SD Median (Q1, Q3)** | **N** | **Mean ± SD Median (Q1, Q3)** | **N** | **Mean ± SD Median (Q1, Q3)** |
| Indexed RV End-diastolic area, cm^2^/BSA^0.8^ | None/Mild TR | 429 | 20.86 ± 4.48 | 339 | 21.84 ± 4.96 | 264 | 25.71 ± 5.42 | 228 | 23.22 ± 4.69 | 175 | 23.39 ± 5.14 | 151 | 22.55 ± 4.57 |
|  |  |  | 20.87 (17.79, 23.70) |  | 21.66 (18.46, 24.48) |  | 25.19 (22.41, 29.06) |  | 23.06 (19.95, 25.90) |  | 22.87 (20.16, 26.25) |  | 22.18 (19.54, 25.73) |
|  | >Mild TR | 56 | 22.11 ± 4.94 | 107 | 22.81 ± 4.95 | 101 | 28.02 ± 7.09 | 66 | 25.81 ± 6.89 | 44 | 26.93 ± 4.92 | 37 | 26.96 ± 6.09 |
|  |  |  | 22.07 (18.51, 26.34) |  | 23.18 (19.32, 26.23) |  | 27.53 (22.51, 31.47) |  | 24.70 (21.45, 29.45) |  | 25.56 (23.63, 29.84) |  | 26.63 (23.23, 30.58) |
|  |  | P = 0.0527 | | P = 0.0784 | | **P = 0.0036** | | **P = 0.0052** | | **P < 0.0001** | | **P = 0.0002** | |
| RV fractional area change | None/Mild TR | 429 | 35.36 ± 8.54 | 339 | 36.96 ± 7.87 | 265 | 33.83 ± 7.41 | 228 | 33.12 ± 6.39 | 175 | 31.44 ± 6.22 | 151 | 33.36 ± 5.56 |
|  |  |  | 35.00 (30.00, 40.00) |  | 37.00 (32.00, 41.00) |  | 33.00 (29.00, 38.00) |  | 33.00 (29.00, 37.00) |  | 31.26 (27.47, 35.41) |  | 33.07 (30.04, 36.70) |
|  | >Mild TR | 56 | 33.75 ± 9.13 | 107 | 35.02 ± 7.37 | 101 | 32.64 ± 8.41 | 66 | 31.09 ± 8.62 | 44 | 30.93 ± 5.36 | 37 | 31.38 ± 5.66 |
|  |  |  | 33.50 (28.00, 40.00) |  | 34.00 (31.00, 40.00) |  | 33.00 (27.00, 38.00) |  | 32.00 (25.00, 37.00) |  | 30.85 (27.38, 34.25) |  | 31.80 (27.92, 35.18) |
|  |  | P = 0.1896 | | **P = 0.0241** | | P = 0.1867 | | P = 0.0787 | | P = 0.8162 | | P = 0.0547 | |
| RV Sphericity Index | None/Mild TR | 458 | 0.80 ± 0.20 | 353 | 0.83 ± 0.20 | 284 | 0.80 ± 0.20 | 250 | 0.85 ± 0.19 | 209 | 0.85 ± 0.18 | 194 | 0.85 ± 0.17 |
|  |  |  | 0.78 (0.65, 0.96) |  | 0.82 (0.67, 0.98) |  | 0.78 (0.64, 0.97) |  | 0.84 (0.71, 1.00) |  | 0.85 (0.70, 1.00) |  | 0.87 (0.71, 1.00) |
|  | >Mild TR | 63 | 0.88 ± 0.20 | 113 | 0.90 ± 0.24 | 102 | 0.80 ± 0.21 | 71 | 0.80 ± 0.18 | 46 | 0.83 ± 0.21 | 41 | 0.93 ± 0.19 |
|  |  |  | 0.89 (0.71, 1.02) |  | 0.88 (0.73, 1.00) |  | 0.78 (0.64, 0.92) |  | 0.78 (0.66, 0.95) |  | 0.80 (0.69, 0.96) |  | 0.96 (0.75, 1.09) |
|  |  | **P = 0.0029** | | **P = 0.0055** | | P = 0.9444 | | **P = 0.0373** | | P = 0.5650 | | **P = 0.0100** | |
| All p-values are t-tests (Pooled or Satterthwaite as appropriate for equality of variance) | | | | | | | | | | | | | |
